# Cytochalasin B Influences Cytoskeletal Organization and Osteogenic Potential of Human Wharton’s Jelly Mesenchymal Stem Cells

**DOI:** 10.3390/ph16020289

**Published:** 2023-02-14

**Authors:** Luca Pampanella, Provvidenza Maria Abruzzo, Riccardo Tassinari, Andrea Alessandrini, Giovannamaria Petrocelli, Gregorio Ragazzini, Claudia Cavallini, Valeria Pizzuti, Nicoletta Collura, Silvia Canaider, Federica Facchin, Carlo Ventura

**Affiliations:** 1 Department of Experimental, Diagnostic and Specialty Medicine (DIMES), University of Bologna, Via Massarenti 9, 40138 Bologna, Italy; 2 Eldor Lab, Via Corticella 183, 40129 Bologna, Italy; 3 Department of Physics, Informatics and Mathematics, University of Modena and Reggio Emilia, Via Campi 213/A, 41125 Modena, Italy; 4 CNR-Nanoscience Institute-S3, Via Campi 213/A, 41125 Modena, Italy; 5 National Laboratory of Molecular Biology and Stem Cell Bioengineering of the National Institute of Biostructures and Biosystems (NIBB) c/o Eldor Lab, Via Corticella 183, 40129 Bologna, Italy

**Keywords:** Cytochalasin B, Wharton’s jelly mesenchymal stem cells, cytoskeleton, osteogenesis, gene expression, atomic force microscope

## Abstract

Among perinatal stem cells of the umbilical cord, human Wharton’s jelly mesenchymal stem cells (hWJ-MSCs) are of great interest for cell-based therapy approaches in regenerative medicine, showing some advantages over other MSCs. In fact, hWJ-MSCs, placed between embryonic and adult MSCs, are not tumorigenic and are harvested with few ethical concerns. Furthermore, these cells can be easily cultured in vitro, maintaining both stem properties and a high proliferative rate for several passages, as well as trilineage capacity of differentiation. Recently, it has been demonstrated that cytoskeletal organization influences stem cell biology. Among molecules able to modulate its dynamics, Cytochalasin B (CB), a cyto-permeable mycotoxin, influences actin microfilament polymerization, thus affecting several cell properties, such as the ability of MSCs to differentiate towards a specific commitment. Here, we investigated for the first time the effects of a 24 h-treatment with CB at different concentrations (0.1–3 μM) on hWJ-MSCs. CB influenced the cytoskeletal organization in a dose-dependent manner, inducing changes in cell number, proliferation, shape, and nanomechanical properties, thus promoting the osteogenic commitment of hWJ-MSCs, as confirmed by the expression analysis of osteogenic/autophagy markers.

## 1. Introduction

Stem cell therapy represents the new medical frontier for the treatment of many diseases, such as multiple sclerosis, osteoarthritis, myocardial infarction, graft-versus-host disease, and many other disorders [1]. In particular, human mesenchymal stem cells (hMSCs) appear to be a compelling tool for clinical applications in regenerative medicine. These cells, isolated from adult tissues and from several fetal and perinatal sources, exhibit a self-renewal ability and can differentiate into multiple specialized cell types. The balance between self-renewal and differentiation defines the fate and the role of hMSCs in the human body [2,3,4]. Moreover, once isolated, the hMSCs must be expanded ex vivo for several passages, allowing to obtain enough cells required for cell-based therapeutic approaches [5,6,7,8], in which the ability of hMSCs to modulate the immune cell functions and their paracrine activity play an important role [9].

Among hMSCs, the ones derived from perinatal tissues, such as umbilical cord and amniotic fluid, seem to be the ideal source for allogenic therapeutic settings [5]. In particular, the umbilical cord provides a great source of hMSCs which can be isolated from both umbilical cord blood or Wharton’s jelly (hWJ-MSCs), the mucoid connective tissue enclosing three umbilical vessels, one vein and two arteries [10]. hWJ-MSCs can be harvested from different portions of the umbilical cord such as the intervascular, perivascular, and sub-amniotic areas with a high recovery efficiency (1 to 5 × 10^4^ cells/cm of the umbilical cord) [11]. Unlike MSCs harvested from adult tissues, such as bone marrow or adipose tissue, the isolation of hWJ-MSCs is a non-invasive procedure with few ethical concerns, since the umbilical cord is usually discarded at birth [12,13]. In addition, hWJ-MSCs, due to their short prenatal life, are spared from DNA damage and pro-aging factors. Moreover, hWJ-MSCs express several embryonic stem cell features, retaining a high telomerase activity [10,14,15], but they do not form teratomas upon transplantation due to a reduced expression of pluripotency genes [16]. Finally, hWJ-MSCs can differentiate towards adipogenic, chondrogenic, and osteogenic cytotypes and retain their stemness properties for several passages in vitro along with a high proliferative rate [5].

Recently, it was discovered that the cytoskeletal network is crucial to direct cell shape, proliferation, senescence, and differentiation by influencing mechano-sensing and mechano-transduction pathways [17,18,19]. Particularly, the cytoskeleton may act on MSC fate, as can be demonstrated by changes in mechanical properties detected during specific commitment of hMSCs [17,20,21,22]. For instance, under adipogenic commitment, MSCs lose and disorganize their cytoskeletal framework, taking a round shape and disassembling the focal adhesions. In contrast, the osteogenic commitment is encouraged by a stiffer and well-organized cytoskeleton as well as the presence of focal adhesions. Moreover, it is known that cytoskeletal relaxation in MSC cultures induces the expression of stemness and pluripotency genes, such as *NANOG*, during the early phase of differentiation [23,24,25,26].

Several molecules may interact with the cytoskeletal framework, inducing changes in its dynamics. Some molecules act favoring the polymerization of cytoskeleton filaments, while others inhibit it [27]. Cytochalasins, first isolated in the 1960s, are a family of 60 mycotoxins produced by different species of fungi able to modify cytoskeletal properties based on their different chemical structures [27,28,29]. Among them, Cytochalasin B (CB) interferes with the entire assembly process of actin microfilaments; by acting on nucleation-elongation and annealing [28,30], CB causes visible changes in cell morphology and shows a direct effect on cellular properties [31,32]. CB interferes with the formation of the mitotic spindle through the destabilization of actin filaments and the inhibition of the cyclin-dependent kinase 1 (CDK1), causing cell cycle arrest in the G2/M transition phase and entry into the G0 resting phase. Once CB is removed, its effects are reversed, allowing the cells to reactivate their cycle by restoring the actin cytoskeleton [33,34]. Moreover, CB inhibits the formation of the actin contractile ring and, consequently, of the cleavage furrow during mitosis. This property prevents cells from accomplishing cytoplasmic division, while nuclei continue to form leading to enlarged and multinucleated cells [28,35]. Finally, CB interferes with cell migration and developmental commitment [24], as well as cell differentiation [22,23,24,25,26]. In our previous research, we demonstrated that CB was able to increase adipogenic commitment in human adipose stem cells (hASCs), modulating their actin organization [22].

In this study we analyzed the cytoskeleton organization and osteogenic potential of hWJ-MSCs subjected to the action of CB, to better characterize this perinatal cellular model and to evaluate the potential use of CB as a tool for controlling hWJ-MSC properties, thus favoring their use in the clinical setting.

hWJ-MSCs were preliminary treated with CB at different concentrations (from 0.1 μM to 3 μM) for 24 h, to analyze CB cytotoxicity and its effects on cell number, proliferation, morphology, and cytoskeletal organization, focusing on actin microfilaments, vinculin, a component of the focal adhesion complexes, and vimentin, a protein belongs to intermediate filaments. Moreover, to deepen the effects of CB on cell cycle and on stemness, the expression pattern of genes involved in both processes was studied. Afterwards, to verify whether a 24 h CB treatment affects hWJ-MSC nanomechanical properties according to the observed morphological and cytoskeletal changes, atomic force microscopy (AFM) analysis was performed. Finally, based upon the close relationship between cell mechanical properties and differentiation [36,37], the effects of CB were evaluated on the ability of hWJ-MSCs to differentiate towards osteogenic lineage.

## 2. Results

### 2.1. hWJ-MSC Characterization

**Figure 1:**
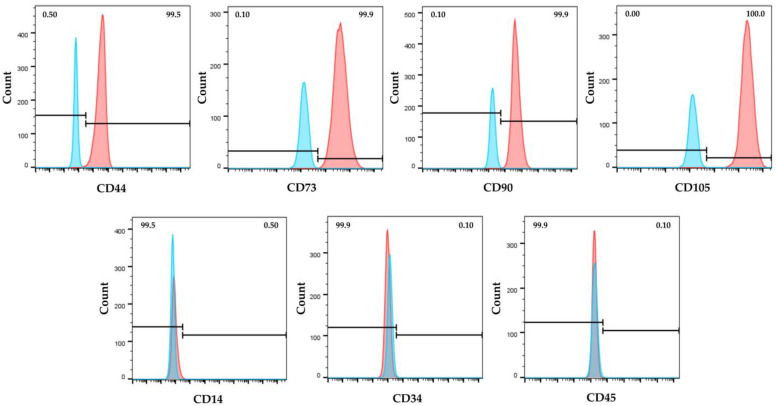
Immunophenotype of human Wharton’s jelly mesenchymal stem cells (hWJ-MSCs) by flow cytometry. Cells were positive for the expression of mesenchymal CD44, CD73, CD90, and CD105 surface markers and negative for hematopoietic CD14, CD34 and CD45 ones. Blue and red histograms represent unstained cells (control) and stained cells with specific antibody, respectively.

**Figure 2:**
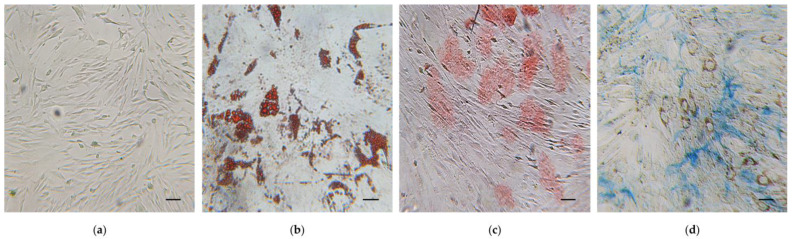
Trilineage differentiation potential of human Wharton’s jelly mesenchymal stem cells (hWJ-MSCs). (**a**) hWJ-MSCs undifferentiated. (**b**–**d**) hWJ-MSCs differentiated in: (**b**) adipocytes with intracellular lipid vacuoles stained in red using Oil Red O (O.R.O) solution; (**c**) osteocytes with calcium deposits in red after Alizarin Red S staining; (**d**) Cartilage proteoglycans of chondrocytes stained with Alcian Blue. Scale bars: 50 μm.

Immunophenotypic analysis, obtained by flow cytometry, showed that hWJ-MSCs expressed the major surface MSC markers (CD44, CD73, CD90, CD105), and were negative for the hematopoietic ones (CD14, CD34 and CD45) (Figure 1). Moreover, isolated cells exhibited the typical MSC fibroblast-like shape (Figure 2a), and they were able to differentiate toward adipogenic, osteogenic, and chondrogenic lineages (Figure 2b–d).

### 2.2. Effects of Cytochalasin B on hWJ-MSC Number and Viability

**Figure 3:**
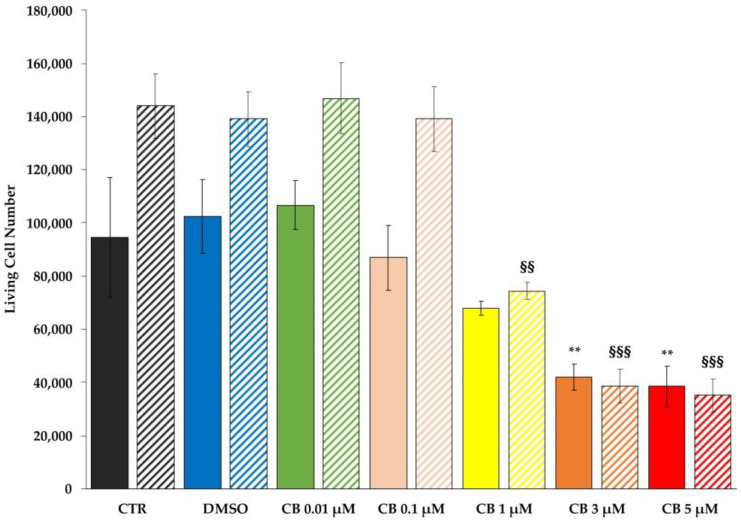
Cell counting of living human Wharton’s jelly mesenchymal stem cells (hWJ-MSCs) after Cytochalasin B (CB) treatment. hWJ-MSCs were seeded at a density of 48,000 cells/well and were maintained for 24 h in standard conditions before treating them with CB (0.01, 0.1, 1, 3 and 5 μM), or dimethyl sulfoxide (DMSO) 0.05% (CB vehicle). Untreated hWJ-MSCs (CTR) were used as control. The count of hWJ-MSC living cell number was assessed after 24 h and 72 h from the beginning of the treatment. Solid- and striped-coloured columns refer to 24 and 72 h-treatment, respectively. Histograms represent the mean of living cell number/well ± standard deviation (SD); *n* = 3. ** *p* < 0.01 vs. 24 h-CTR cells; ^§§^ *p* < 0.01 and ^§§§^ *p* < 0.001 vs. 72 h-CTR cells.

To evaluate the effects of different CB concentrations (range 0.01–5 μM) on hWJ-MSC number, cells were counted 24 h and 72 h after CB treatment. At both experimental time points, the number of living cells was reduced in a dose-dependent manner (Figure 3). In particular, while hWJ-MSCs treated with CB 0.01, 0.1 μM or with the CB vehicle dimethyl sulfoxide (DMSO) showed no differences in cell number compared to untreated cells (CTR), hWJ-MSCs treated with the highest concentrations of CB showed a statistically significant reduction in cell number; the extent of such reduction was similar between the treatment of 3 and 5 μM CB (Figure 3). Based on these preliminary results, in the following experiments we decided to investigate the effects of CB on hWJ-MSC properties only at 0.1, 1 and 3 μM.

**Figure 4:**
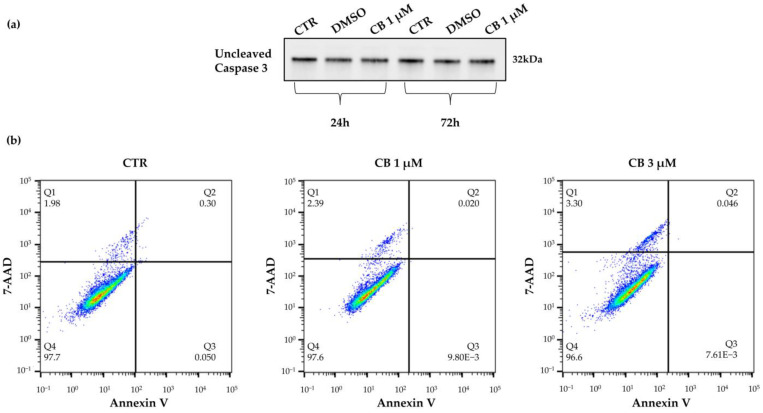
Viability of human Wharton’s jelly mesenchymal stem cells (hWJ-MSCs) after a 24 h-Cytochalasin B (CB) treatment. (**a**) Uncleaved (total) caspase 3 expression was investigated in untreated (CTR) or in treated hWJ-MSCs with CB 1 μM or dimethyl sulfoxide (DMSO) 0.05% (CB vehicle) for 24 h or 72 h. (**b**) hWJ-MSC viability was assessed using the Annexin V-7AAD staining assay. hWJ-MSCs were treated for 24 h without (CTR) or with CB (1 and 3 μM). Quadrants (Q) represent the percentage of necrotic cells (Q1), late apoptotic cells (Q2), early apoptotic cells (Q3) and living cells (Q4).

To investigate whether the reduction in cell number was due to a cytotoxic effect of CB, the expression of uncleaved (total) caspase 3, a marker of apoptosis, was evaluated after cells were treated with CB 1 μM for 24 and 72 h by Western blot. As shown in the Supplementary Figure S1, the amount of total proteins loaded on SDS-PAGE gel was found to be comparable between samples. No difference in caspase 3 expression was observed between treated and untreated cells (CTR) at both investigated time points, demonstrating that CB did not increase apoptosis even after a prolonged CB exposure (Figure 4a).

These results were further supported by the Annexin V-7AAD Staining Assay which allows to distinguish the percentage of living cells from apoptotic and necrotic cells. hWJ-MSCs treated with CB 1 μM for 24 h retained high viability (>95%) compared to CTR (Figure 4b, lower left quadrant, Q4), indicating that the reduction in cell number was not directly linked to cell death. In fact, hWJ-MSCs treated with CB were negative for Annexin V staining, a marker of apoptosis (upper and lower right quadrants, Q2 and Q3) and showed a similar percentage of 7AAD necrotic cells (upper left quadrant, Q1) compared to CTR (Figure 4b). Similar results were obtained when the highest CB concentration, 3 μM, was used (Figure 4b).

### 2.3. Effects of Cytochalasin B on hWJ-MSC Proliferation and Cell Cycle Progression

To assess whether the reduction in cell number was linked to a decrease in cell proliferation, the hWJ-MSCs were incubated with a 5-Bromo-2′-deoxyuridine (BrdU) labelling solution after treatment with CB (0.1, 1 and 3 μM) or DMSO for 24 h or 72 h.

**Figure 5:**
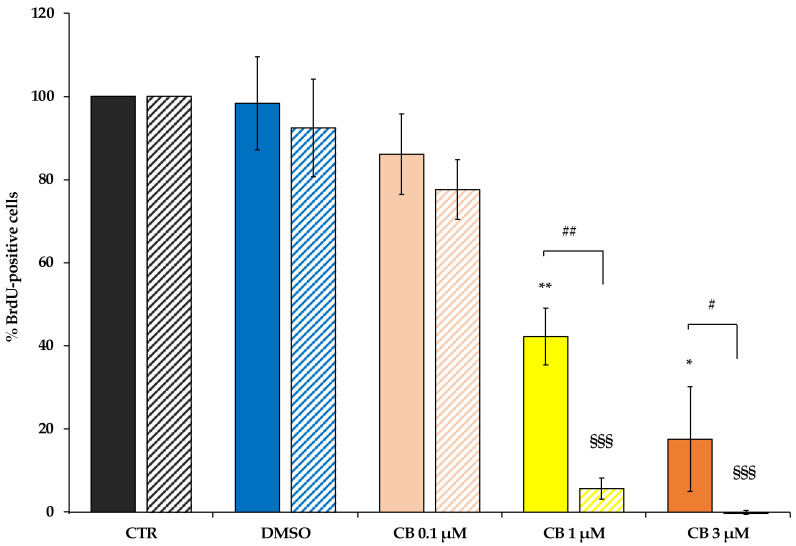
Proliferation ability of human Wharton’s jelly mesenchymal stem cells (hWJ-MSCs) after 24 h- and 72 h-Cytochalasin B (CB) treatment. hWJ-MSC proliferation ability was assessed in untreated (CTR) and in CB (0.1, 1 and 3 μM) or in dimethyl sulfoxide (DMSO) 0.05% (CB vehicle) treated cells after a 24 h- (solid-coloured columns) or 72 h-treatment (coloured and striped columns). Histograms represent the mean percentage of BrdU incorporation ± standard deviation (SD); *n* = 3; * *p* < 0.05 and ** *p* < 0.01 vs. 24 h-CTR cells; ^§§§^ *p* < 0.001 vs. 72 h-CTR cells; ^#^ *p* < 0.05 and ^##^ *p* < 0.01 24 h- vs. 72 h-treated cells.

The assessment of cell proliferation is based on BrdU incorporation, a thymidine analogue, in the newly synthesized DNA strands in dividing cells. At both experimental time points, CB treatment decreased the percentage of BrdU positive cells in a dose-dependent manner compared to CTR. Such reduction reached statistical significance starting from CB 1 μM, while DMSO did not affect hWJ-MSC proliferation (Figure 5). Moreover, at 72 h the effects of CB were more pronounced compared to 24 h-treated cells; in fact, at 72 h the percentage of BrdU incorporation was strongly decreased in cells treated with both 1 and 3 μM CB, suggesting that, at this experimental time-point, proliferation was abrogated even at the lower CB concentration (Figure 5).

**Figure 6:**
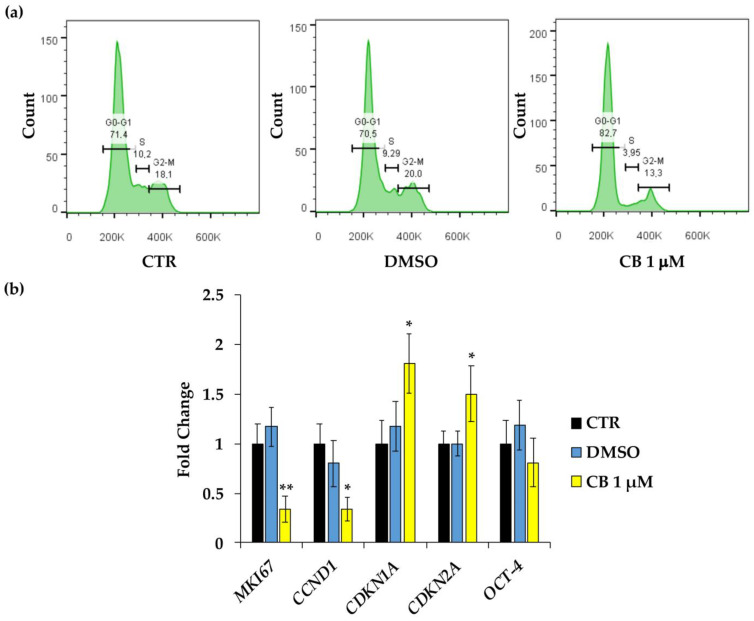
Cell cycle control in human Wharton’s jelly mesenchymal stem cells (hWJ-MSCs) after a 24 h-Cytochalasin B (CB) treatment. (**a**) Cell cycle analysis was assessed by flow cytometry in hWJ-MSCs treated with CB 1 μM, dimethyl sulfoxide (DMSO) 0.05% (CB vehicle), or without treatment (CTR) for 24 h. The quantification of hWJ-MSCs in each cell cycle phase gate (G1/G0, S, and G2/M) is presented as percentage of cells. (**b**) The expression of *MKI67* (*Proliferation marker protein Ki-67*), *CCND1* (*cyclin D1*), *CDKN1A* (*cyclin dependent kinase inhibitor 1A*), *CDKN2A* (*cyclin dependent kinase inhibitor 2A*) and *OCT-4* (*Octamer-binding transcription factor 4*) genes was assessed in hWJ-MSCs treated with CB 1 μM, DMSO, or without treatment (CTR) for 24 h. Data were normalized using three housekeeping genes (*glyceraldehyde 3-phosphate dehydrogenase*—*GAPDH*, *TATA box binding protein*—*TBP* and *tyrosine 3 monooxygenase/tryptophan 5-monooxygenase activation protein zeta*—*YWHAZ*); the normalized expression value of CTR was set up to 1, and all other gene expression values were reported to that sample. Data are reported as normalized fold change ± standard deviation (SD); *n* = 3. Black columns refer to CTR cells, blue columns refer to DMSO-treated cells, yellow columns refer to CB-treated cells. * *p* < 0.05 and ** *p* < 0.01 vs. CTR.

Moreover, to confirm the proliferation data and to verify whether CB interferes with cell cycle progression, flow cytometry analysis was performed. Compared to CTR cells, the treatment of hWJ-MSCs with CB 1 μM increased the percentage of cells in the G0/G1 phase and led to a drop in the percentage of cells in S and in G2/M phases (Figure 6a).

To confirm cell cycle block, the expression of genes involved in cell proliferation and cell cycle control were investigated in hWJ-MSCs treated for 24 h only with CB 1 μM; at this concentration, the effects of CB on cell proliferation started to be evident. CB induced a significant reduction in the expression of *proliferation marker protein Ki-67* (*MKI67*) and *cyclin D1* (*CCND1*), and a parallel increase in *cyclin dependent kinase inhibitor 1A* (*CDKN1A*, alias *p21*) and *cyclin dependent kinase inhibitor 2A* (*CDKN2A*, alias *p16^INK4α^*) mRNA abundance compared to the CTR (Figure 6b). Whereas, to investigate the role of CB on cell stemness, the expression of the *Octamer-binding transcription factor 4* (*OCT-4* or *POUF51A*) was evaluated, demonstrating that CB did not interfere with its expression. hWJ-MSCs treated with DMSO showed no differences in gene expression compared to CTR for all the investigated target genes.

### 2.4. Effects of Cytochalasin B on hWJ-MSC Migration Ability

**Figure 7:**
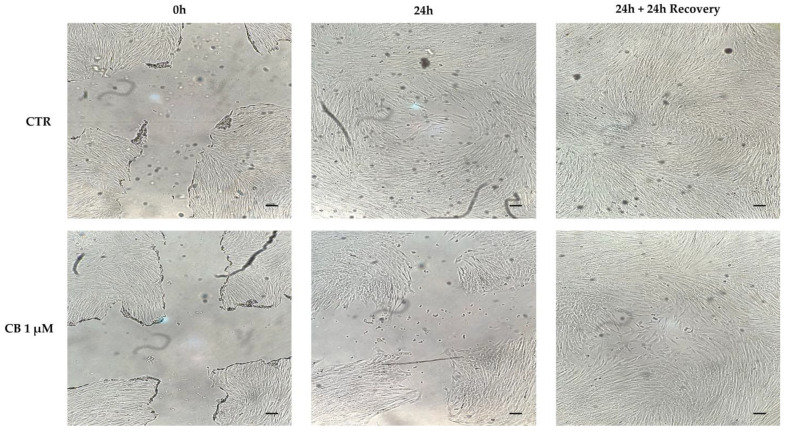
Cytochalasin B (CB) effects on human Wharton’s jelly mesenchymal stem cells (hWJ-MSCs) migration ability using the scratch wound assay. The ability of cells to close the wound in untreated (CTR) and in CB 1 μM-treated cells were analyzed after a 24 h-treatment (24 h) and after a 24 h-recovery (24 h + 24 h recovery) time from CB removal. Images were acquired immediately after scratch (0 h) and at the indicated time-points (24 h and 24 h + 24 h recovery) using the Leica Labovert FS Inverted Microscope equipped with a Leica MC170 HD Imaging System Camera. Scale bars: 50 μm.

To evaluate the migratory capacity of hWJ-MSCs after treatment with CB, the scratch wound assay was performed. As shown in Figure 7, a 24 h-CB treatment decreased the ability of hWJ-MSCs to close the scratch “wound” compared to CTR (Figure 7). These findings were consistent with the reduction in hWJ-MSC proliferation induced by CB that consequently slowed hWJ-MSC migration ability.

At the same time, at 24 h from CB removal, hWJ-MSCs restored their proliferative ability and thus, they closed the initial wound, confirming the reversible effects of CB previously described [22,27].

### 2.5. Effects of Cytochalasin B on hWJ-MSC Morphology

**Figure 8:**
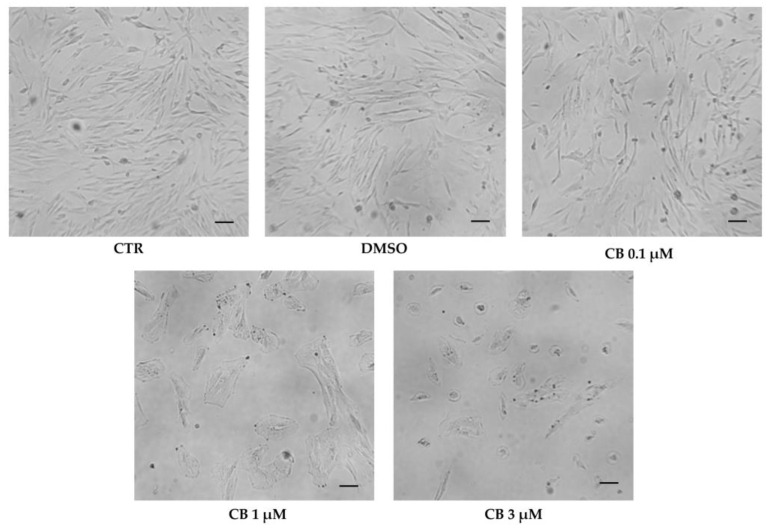
Morphology of human Wharton’s jelly mesenchymal stem cells (hWJ-MSCs) after 24 h in the presence of Cytochalasin B (CB) 0.1 μM, 1 μM, and 3 μM, dimethyl sulfoxide (DMSO) 0.05% (CB vehicle) or without treatment (CTR). Images were acquired using the Leica Labovert FS Inverted Microscope equipped with a Leica MC170 HD Imaging System Camera. Scale bars: 50 μm.

Cell morphology was evaluated in untreated (CTR) and in CB- or DMSO-treated hWJ-MSCs after 24 h of exposure. DMSO treatment did not influence cell shape. The alterations of cell morphology emerged progressively upon increasing the CB concentration. hWJ-MSCs treated with CB 1 μM showed an enlarged shape and cells appeared spreading on the culture support. At concentration of 3 μM CB, cells became more rounded losing their fibroblast-like shape (Figure 8).

### 2.6. Effects of Cytochalasin B on the Localization and Expression of Cytoskeletal Markers in hWJ-MSCs

**Figure 9:**
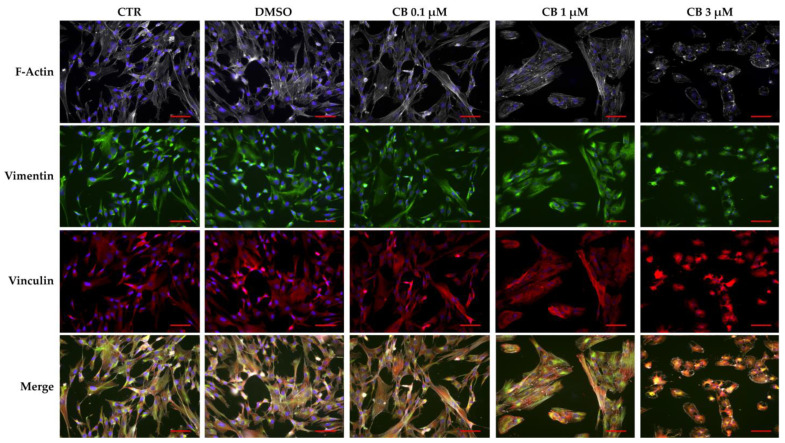
Immunofluorescence of human Wharton’s jelly mesenchymal stem cell (hWJ-MSC) cytoskeletal markers after 24 h of Cytochalasin B (CB) treatment. hWJ-MSCs were immunostained with Phalloidin (grey signal, specific for F-Actin), anti-vimentin (green signal), or anti-vinculin (red signal) after 24 h of dimethyl sulfoxide (DMSO) 0.05% (CB vehicle) or CB (0.1, 1, or 3 μM) exposure. Untreated cells were used as CTR. NucBlue^®^ Fixed Cell ReadyProbes^®^ Reagent (DAPI) was used to counter-stain nuclei (blue signal). Images were acquired using a Nikon inverted microscope Eclipse Ti2-E and a digital sight camera DS-Qi2, through the imaging software NIS-Elements. Scale bars: 50 μm.

To investigate the effects of CB on cytoskeletal organization, hWJ-MSCs untreated (CTR) and treated with 0.1, 1 and 3 μM CB or DMSO for 24 h, were immunostained with Phalloidin (specific for F-Actin), anti-vinculin or anti-vimentin antibodies (ABs). As shown in Figure 9, CB induced a dose-dependent cytoskeletal reorganization; actin stress fibers (grey signal) were still evident up to the concentration of 1 μM. On the contrary, at 3 μM CB, actin fibers remained localized only at cortical level to support the cell structure (Figure 9 and Figure S2 upper panel). At the same time, the distribution and/or organization of vimentin (green signal) changed when hWJ-MSCs were treated with CB 3 μM; at this concentration vimentin formed clusters, following the stress fiber reorganization (Figure 9 and Figure S2 middle panel). Finally, CB modified the vinculin distribution in a dose-dependent manner showing at least at 1 μM CB its involvement in focal adhesion formation (Figure 9). Moreover, multinucleated cells appeared with the increasing concentration of CB (Figure S2 lower panel).

### 2.7. Effects of Cytochalasin B on the Nanomechanical Properties of hWJ-MSCs

The effects produced by CB on the mechanical properties of hWJ-MSCs were evaluated with AFM, which allows to study the changes in elasticity and viscosity in single cells at the nano-scale level.

To obtain the complete viscoelastic characterization of untreated hWJ-MSCs (used as CTR) or hWJ-MSCs cultured in the presence of CB (0.1, 1, and 3 μM CB) after 24 h of treatment, Ting’s model associated with the power-law-relaxation (PLR) process was applied to the approach/retract profiles of the force curves [38]. In this model, viscosity is represented by the α power-law exponent (α = 0 indicates a completely elastic behavior; α = 1 indicates a completely fluid behavior), whereas the cell elastic stiffness component is given by *E*_0_—i.e., the initial Young’s modulus felt by the cantilever tip upon contact with the sample.

**Figure 10:**
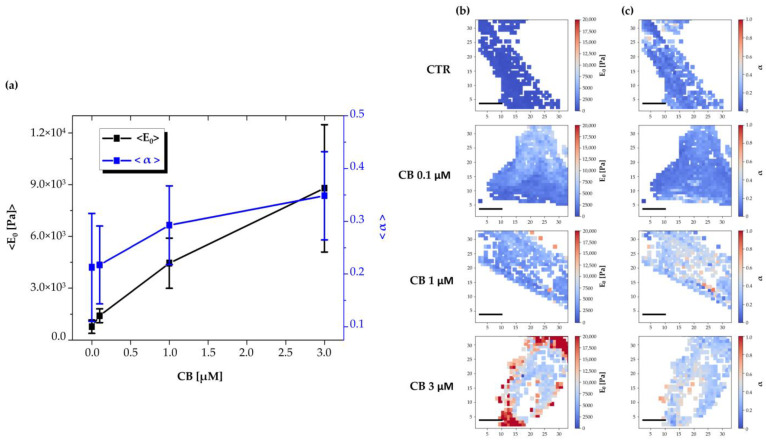
Evaluation of changes in human Wharton’s jelly mesenchymal stem cells (hWJ-MSCs) viscoelasticity during the treatment with different concentrations of Cytochalasin B (CB) using Ting’s model. (**a**) Time-wise behavior for the representative Young’s modulus (*E*_0_) and power-law exponent (α) values in the untreated (CTR) and CB-treated cells (0.1, 1, and 3 μM). The continuous lines are guides for the eye. Images are representative stiffness (**b**) and viscosity (**c**) maps of single untreated (CTR) or treated cells with CB at 0.1, 1, and 3 μM. Each point in a map refers to a specific value of the Young’s modulus (*E*_0_) or of the power-law exponent (α) and refers to a false color scale. The color scale is shown to the right of each row. The white regions around the cell represent the substrate (the white dots within the cell regions are due to individual force curves that have been discarded due to poor quality). Scale bars: 20 μm.

As shown in Figure 10a, results obtained by the analysis of stiffness and viscosity data using Ting’s model, revealed that after 24 h of CB treatment a dose-dependent enhancement in the *E*_0_ elastic modulus was found, suggesting a major cell stiffness; for the viscosity related parameter (α), an increasing trend was observed, suggesting a tendency of cells to become more fluid. In Figure 10b,c, representative stiffness and viscosity maps of single cells are shown (untreated or treated with 0.1, 1, and 3 μM CB, respectively). Red points indicate higher *E*_0_ or α values, and blue points indicate lower values. Noteworthy, a major stiffness at the cell peripheral level was evident at 3 μM CB.

### 2.8. Effects of Cytochalasin B on the Osteogenic Potential of hWJ-MSCs

Before starting the osteogenic induction protocol, the effects of CB on hWJ-MSC viability were evaluated by counting cells after 21 days of CB treatment. hWJ-MSC number was reduced in a dose-dependent manner and cells showed more than 85% viability even when the highest concentration of CB, 3 μM, was used (Figure S3).

Then, the effects of CB on hWJ-MSC osteogenic commitment were studied during the entire 21 day-osteogenic differentiation period. Cells were cultured in the standard or in osteogenic differentiation medium, without (CTR) or with DMSO or CB at 0.1, 1, or 3 μM.

**Figure 11:**
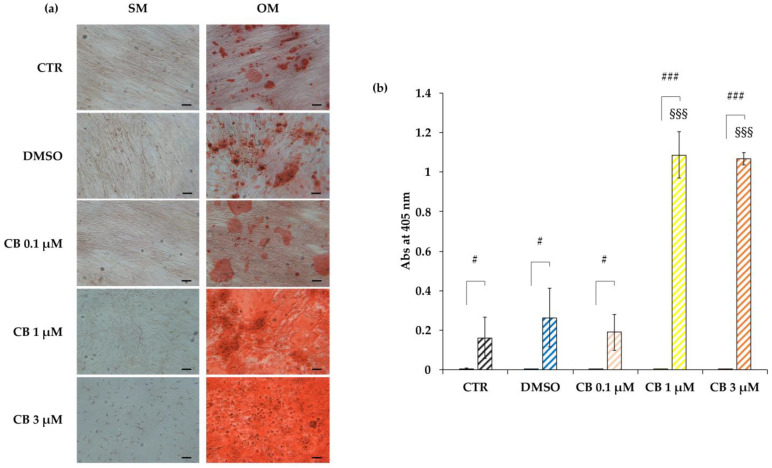
Osteogenic potential of human Wharton’s jelly mesenchymal stem cells (hWJ-MSCs) treated with Cytochalasin B (CB). hWJ-MSCs were induced to differentiate into the osteogenic lineage for a total period of 21 days. Cells were cultured in standard medium (SM) or in osteogenic differentiation medium (OM), without (CTR) or with dimethyl sulfoxide (DMSO) 0.05% (CB vehicle) or CB at 0.1, 1, or 3 μM. The final osteogenic commitment was evaluated by Alizarin Red S staining. (**a**) Representative images of hWJ-MSCs cultured in SM or in OM and stained with Alizarin Red S, were acquired under phase contrast illumination using a light microscope (Leica Labovert FS Inverted Microscope) and a Leica MC170 HD Imaging System Camera. Osteogenesis was highlighted by the presence of red calcium deposits. Scale bars: 50 μm. (**b**) Left columns: hWJ-MSCs cultured in SM; coloured and striped right columns: hWJ-MSCs cultured in OM. The Alizarin Red S dye incorporated into the matrix was solubilized with 10% acetic acid and absorbance was read at 405 nm with a spectrophotometer plate reader (Wallac 1420 Victor2 Multilabel Counter). Data are expressed as mean of Alizarin Red S absorbance (Abs) at 405 nm ± standard deviation (SD). ^#^ *p* < 0.05 and ^###^ *p* < 0.001 vs. corresponding hWJ-MSCs cultured in SM; **^§§§^** *p* < 0.001 vs. untreated hWJ-MSCs (CTR) cultured in OM; *n* = 3.

Cells cultured in the osteogenic medium differentiated into osteocytes in all investigated conditions, highlighting the great efficiency of the induction program. Moreover, CB influenced the osteogenic commitment of hWJ-MSCs in a dose-dependent manner: in particular, the Alizarin Red S staining highlighted an increase in calcium deposits in hWJ-MSCs treated with CB at 1 and 3 μM compared with CTR and DMSO-treated cells, suggesting that CB enhanced the osteogenic potential of hWJ-MSCs (Figure 11a). These data were confirmed by the measurement of the absorbance at 405 nm of the Alizarin Red S dye extracted from both non-induced and induced samples (Figure 11b). Moreover, the obtained data indicated that CB alone was not able to induce the osteogenic commitment and that no difference in osteogenic differentiation was observed between CTR and DMSO-treated hWJ-MSCs (Figure 11). For these reasons, DMSO-treated hWJ-MSCs were used as control in the following Alizarin Red S staining and gene expression experiments.

**Figure 12:**
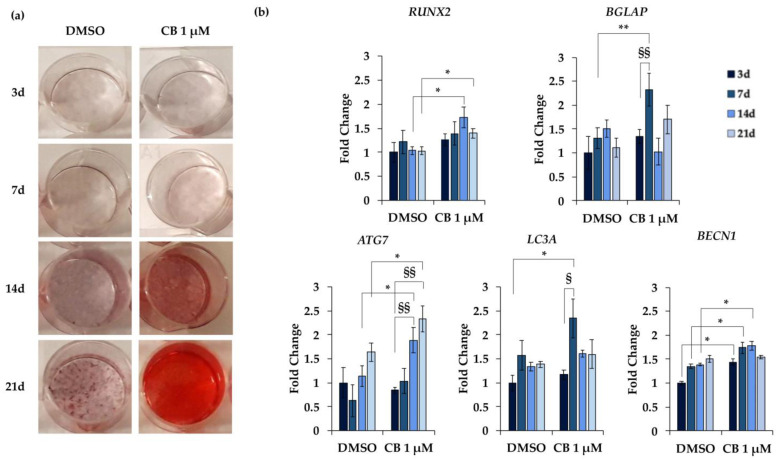
Alizarin Red S staining and gene expression in human Wharton’s jelly mesenchymal stem cells (hWJ-MSCs) during osteogenic differentiation. hWJ-MSCs were cultured in osteogenic medium for 3, 7, 14, and 21 days (d) and were treated with Cytochalasin B (CB) 1 μM or dimethyl sulfoxide (DMSO) 0.05% (CB vehicle). (**a**) Representative images of wells in which hWJ-MSCs were stained with Alizarin Red S. Osteogenesis was highlighted by the presence of the red calcium deposits. (**b**) Expression of *RUNX2* (*RUNX family transcription factor 2*), *BGLAP* (*bone gamma-carboxyglutamic acid-containing protein*, also named *osteocalcin*), *ATG7* (*autophagy related 7*), *LC3A* (*microtubule associated protein 1 light chain 3 alpha*) and *BECN1* (*Beclin 1*) genes was assessed. Data, obtained with real-time PCR, were normalized using three housekeeping genes *(hypoxanthine phosphoribosyl transferase 1*—*HPRT1, TATA box binding protein—TBP and tyrosine 3 monooxygenase/tryptophan 5-monooxygenase activation protein zeta*—*YWHAZ*); the normalized expression value of DMSO after 3 days from the beginning of the differentiation protocol was set up to 1, and all other gene expression values were reported to that sample. Data are reported as normalized fold change ± standard deviation (SD); *n* = 3. * *p* < 0.05 or ** *p* < 0.01 vs. DMSO-treated hWJ-MSCs at the same day; ^§^ *p* < 0.05 or ^§§^ *p* < 0.01 vs. hWJ-MSCs treated with CB 1 μM at 3 days from the beginning of the osteogenic differentiation.

In particular, the osteogenesis was deeply evaluated at 3, 7, 14, and 21 days from the beginning of the osteogenic induction by analyzing the Alizarin Red S staining and by measuring the expression of specific marker genes. For this purpose, hWJ-MSCs were treated with CB 1 μM or DMSO, since the increase in alizarin staining was already evident at CB 1 μM (Figure 12a). CB showed a time-dependent increase in the calcium deposits compared to DMSO-treated cells, confirming that CB enhanced the osteogenic potential of hWJ-MSCs (Figure 12a). Moreover, compared to DMSO-treated cells, CB influenced the expression of *RUNX2* (*RUNX family transcription factor* 2) and *BGLAP* (*bone gamma-carboxyglutamic acid-containing protein*, also named *osteocalcin*), early and late markers of osteogenic differentiation, respectively. In detail, CB-treated cells showed an increased expression of *RUNX2* and *osteocalcin* compared to DMSO-treated cells at both 14 and 21 days and at 7 days of the differentiation program, respectively (Figure 12b).

Moreover, the expression of three marker genes involved in the autophagy process was investigated along the osteogenic differentiation: *ATG7* (*autophagy related 7*), *LC3A* (*microtubule associated protein 1 light chain 3 alpha*), and *BECN1* (*Beclin 1*). Data reported in Figure 12b showed a trend toward increased expression of *ATG7*, *LC3A* and *BECN1* in both DMSO- and CB-treated cells along the differentiation period, reaching statistical significance only in CB-treated cells. These data suggested that the autophagic pathway is involved in osteogenic differentiation. Moreover, compared to DMSO, CB treatment significantly increased the expression of *LC3A* at 7 days, *BECN1* at 3, 7, and 14 days, and *ATG7* at 14 and 21 days, from the beginning of the osteogenic differentiation (Figure 12b). Finally, the expression of all investigated genes increased in hWJ-MSCs cultured in osteogenic medium compared to cells cultured in standard medium, thus confirming the differentiation and the involvement of these marker genes in the osteogenic program (data not shown).

**Figure 13:**
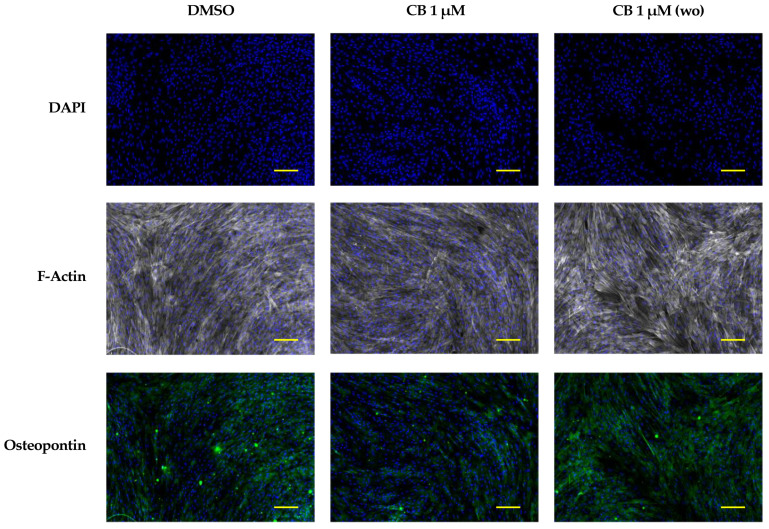
Immunofluorescence analysis in human Wharton’s jelly mesenchymal stem cells (hWJ-MSCs) of cytoskeletal and osteogenic markers at 7 days from the beginning of the osteogenic program. hWJ-MSCs were cultured in osteogenic medium with dimethyl sulfoxide (DMSO) 0.05% (CB vehicle) or CB 1 μM for the entire experimental time (continuous CB). hWJ-MSCs were also cultured in osteogenic medium (OM) in presence of CB 1 μM for only 24 h, and then removed, and cells were cultured in OM up to the end of the experiment (wo = CB wash-out). hWJ-MSCs were immunostained with Phalloidin (grey signal, specific for F-Actin) or anti-osteopontin (green signal) at 7 days from the beginning of the osteogenic program. NucBlue^®^ Fixed Cell ReadyProbes^®^ Reagent (DAPI) was used to counter-stain nuclei (blue signal). Images were acquired using a Nikon inverted microscope Eclipse Ti2-E and a digital sight camera DS-Qi2, through the imaging software NIS-Elements. Scale bars: 100 μm.

To strengthen the hypothesis that CB, influencing the cytoskeleton organization, promotes the osteogenic program, we performed an osteogenic differentiation experiment in which CB was washed-out after 24 h of treatment. Cytoskeletal and osteogenic markers were analyzed at 7 days from the beginning of the osteogenic program. As shown in Figure 13, when CB was removed from the osteogenic medium, the organization of F-Actin and the expression of osteopontin in CB-treated hWJ-MSCs were comparable to those of the DMSO control sample; on the contrary, the presence of CB in the osteogenic medium for the entire period of differentiation induced alteration in the F-Actin distribution and a decrease in osteopontin expression, indicating a clear relationship between cytoskeleton organization and the expression of osteogenic markers (Figure 13).

## 3. Discussion

It is known that the cytoskeletal dynamics play a pivotal role in directing cell properties as proliferative and differentiative potentials. Therefore, the modulation of the cytoskeletal organization may help at leading to a specific cell fate [17,20], allowing the use of several MSC-types for particular cell-based therapies.

Several molecules may influence the cytoskeletal framework, interacting specifically with one of the three cytoskeletal filaments or their associated proteins [27]. Cytoskeletal inhibitors may either disrupt (e.g., cytochalasins and vincristine) or rigidify (e.g., jasplakinolide and paclitaxel) actin microfilaments and microtubules [28]. Among all cytochalasins, Cytochalasin B (CB) and Cytochalasin D (CD) have been the most studied. Although CB and CD interfere with actin fiber polymerization, CD showed higher potency to disrupt the actin cytoskeleton compared to CB, and this difference can be explained by the different chemical structures of the two molecules [39]. Moreover, unlike CD, CB has been shown to inhibit the transport of glucose [40]. The different properties of CB and CD may specifically affect cellular behavior and stem cell fate.

Since it is known that the effects of cytochalasins depend not only on the type of cytochalasin, but also on the cell-type chosen [41], in this study we investigated, for the first time, the effects of CB on human perinatal mesenchymal stem cells isolated from the Wharton’s jelly of the umbilical cord (hWJ-MSCs). These cells exhibit well known advantages with respect to adult-derived ones: they are abundant, safe, non-invasively harvested after birth, and they show an immunomodulatory activity reducing the probability of rejection after transplantation [42].

Our data collectively showed that CB affects several properties of hWJ-MSCs, such as morphology, proliferation, and osteogenic commitment as revealed by changes in cytoskeletal organization and mechanical properties.

hWJ-MSCs treated with CB for 24 h lost the fibroblast-like shape and showed deep alterations in their morphology in a dose-dependent manner. Cells treated with CB at 1 μM showed an enlarged shape and were largely spread on their plastic support. This cell behavior could be explained by the maintenance of actin filaments and by the presence of large focal adhesion regions connecting the stress fibers to the support, as evidenced by vinculin staining; moreover, cells showed a wider distribution of the intermediate vimentin filaments, towards all cell directions to support both the cytoplasm and the nucleus. At the highest CB concentration (3 μM) the hWJ-MSCs became more rounded due to a deep alteration of cytoskeleton organization: actin microfilaments were distributed at the cortical level, and vinculin assumed a less organized distribution pattern. Vimentin formed intense clusters especially at the perinuclear level, probably forming a cage which could maintain the structural integrity of the nucleus, counteracting cytoskeletal deformations [43]. Thus, we hypothesized that the perinuclear vimentin observed after CB treatment, protecting the nucleus, influenced cell fate and genome expression. Overall morphological and cytoskeletal results demonstrated that the CB effects were not only reversible, but changed in a dose-dependent manner, as already reported for CD: CD at low concentrations promotes actin depolymerization, while CD at higher concentrations can lead to the absence of depolymerization or increased polymerization [41,44].

The cytoskeletal remodeling induced by CB involved changes in the nanomechanical properties of hWJ-MSCs. In fact, our AFM investigation showed that CB induced a dose-dependent increase in cell stiffness (as demonstrated by analysis of *E*_0_ value). hWJ-MSCs treated with CB at 1 μM resulted in increased rigidity, in accordance with the large presence of actin bundles, while cells treated with CB at 3 μM showed an evident major stiffness at the peripheral cell area (see Figure 10b), probably due to the maintenance and strengthening of actin tension. Moreover, hWJ-MSCs showed a trend towards an increase in viscosity dependent on CB concentration. The variation of the viscosity we observed was probably due to a fragmentation of the actin cytoskeleton and to an increased presence of actin fragments inside the cell cytoplasm. It cannot be excluded that the stiffness and cellular viscosity depend on the whole reorganization of other actin binding proteins (ABP) or other cytoskeletal filaments.

Moreover, CB decreased the proliferative potential of the hWJ-MSCs in a dose- and time-dependent manner without affecting cell viability. The effect of CB on proliferation resulted in the reduced ability of hWJ-MSCs to close the scratch, and was recovered after CB removal from the culture medium, highlighting the reversible effect of CB, previously described [22].

To obtain a more complete frame about the effects of CB on hWJ-MSC proliferation and cell cycle regulation, we performed a flow cytometry analysis of the hWJ-MSCs after 24 h of CB treatment. Treated hWJ-MSCs accumulated in G0/G1 phase, and consequently a low percentage of cells in S phase was found. Overall, these data suggest an arrest of the cell cycle.

To confirm this hypothesis, the expression analysis of genes involved in these pathways (*MKI67*, *CCND1*, *p21,* and *p16^INK4α^*), was performed. CB induced a decrease in the gene expression of the cell proliferation marker, *MKI67*, and of *CCND1*, encoding for Cyclin D1, and an increase in *p16^INK4α^* and *p21* gene expression, while the mRNA abundance of *OCT-4*, a stemness marker, was unchanged.

The progression of the cell cycle is modulated by proteins, namely Cyclins, which bind the cyclin-dependent kinases (CDK), forming a complex to exert their functions. In particular, Cyclin D1 is expressed during the transition between G1 and S phases and represents the most pivotal point for cell cycle regulation. p16 and p21 act as CDK inhibitors (CDKI) and are involved in cell cycle arrest. p16 belongs to the INK4 family of CDKI [45] and blocks cell cycle progression by preventing the formation of the CDK4/CDK6-Cyclin D1 complex [46]. Even p21 induces a G1 phase block, preventing the entry in the S phase of cell cycle [46].

Moreover, it is known that both proteins are involved in senescence processes [46]. In particular, p16 is activated by external stressors, such as chemicals and stress culture conditions, namely, senescence-inducing stimuli [47]. p16-induced senescence is referred to as premature senescence, which is associated with morphological changes, an increase of SA-β-gal activity, a reduction of expression of stemness genes, and cell cycle arrest [7,48,49]. On the other hand, p21 is a crucial transcription target of p53 which is necessary for p53-dependent senescence, and its up-regulation is protective against tumor transformation, preventing proliferation of cells with severely impaired DNA [46,47,50].

Although the increase in p16 and p21 are often used to identify cells with senescence-associated phenotypes, it was demonstrated that both proteins might act in a classical way as cell cycle inhibitor as a pre-requisite for differentiation [51]. Indeed, the mechanisms underlying senescence are interconnected with mechanisms involved in cell commitment and into the maintenance of long-term post-mitotic differentiation [52]. For example, hMSCs, positive to senescent markers, showed an increased propensity to osteogenic commitment, as can be inferred by changes in the expression of various differentiation regulatory factors and by a specific cytoskeletal organization [5,7].

Therefore, the reduction in cell proliferation favored by the increased expression of the cell cycle inhibitors p16 and p21, associated with the increased stiffness in CB-treated hWJ-MSCs (underlined by the increased *E*_0_ value), and the presence of focal adhesion and actin bundles may mark the propensity of these cells to differentiate toward the osteogenic pathway. This hypothesis was investigated by comparing the alizarin staining and the gene expression during the osteogenic differentiation protocol in CB-treated and untreated cells. CB increased osteogenic differentiation as demonstrated by the dose-dependent enhancement in calcium deposits and by the expression of the osteogenic markers *RUNX2* and *BGLAP*, throughout the whole differentiation process in the induced cells.

To confirm that osteogenesis in hWJ-MSCs was affected by CB-induced cytoskeletal changes, a wash-out experiment was performed. After CB removal, actin organization and osteopontin expression of hWJ-MSCs cultured in osteogenic medium were similar to those of DMSO control cells, suggesting a clear relationship between CB-induced cytoskeleton alteration and the expression of osteogenic markers. Our results are consistent with data previously reported. For example, Higuchi et al. demonstrated that the effect of another cytochalasin, CD, on cytoskeleton and osteogenic differentiation disappeared within 1 h after CD washout [53]. Moreover, we previously reported that in the hASC model the alterations of cytoskeletal organization induced by CB were closely associated with the stimulation of another differentiation program, adipogenesis, as demonstrated by the increase in the expression of adipogenic markers (i.e., perilipin) and in lipid vacuole content [22]. The decrease in osteopontin levels observed in CB-treated cells revealed the multifaceted features of this sialoprotein during osteogenic differentiation. Although osteopontin is thought to be a marker of osteogenesis which increases during the osteogenic process, some authors demonstrated that this protein acts as a negative regulator of proliferation and differentiation in pre-osteoblastic cells causing, when overexpressed, a decrease in mineral deposition; this evidence suggested that a fine tuning of its expression is necessary during osteogenic differentiation [54]. However, further experiments will be necessary to clarify the role of osteopontin and to strengthen the relationship between the CB effects on cytoskeleton and cell differentiation in mesenchymal stem cell models.

In this study, we also demonstrated that CB increased the expression of *ATG7*, *LC3A,* and *BECN1* encoding proteins involved in the autophagosome formation during autophagy process [55,56]. It was known that autophagy plays a role in maintaining the stemness properties and it is modulated during stem cell differentiation [57]. Several lines of evidence suggest that autophagy drives the osteogenic differentiation [58]. For example, it was demonstrated that the inhibition of autophagy precluded the osteogenic differentiation in human gingival mesenchymal stem cells [59]. Thus, in our study, the activation of the autophagic pathway could be one explanation for the increased propensity to osteogenesis of CB-treated hWJ-MSCs. In fact, the CB inhibition of glucose transporter and the consequent lowest ability to intake glucose, previously described in the literature, could activate autophagy via AMP-activated protein kinase (AMPK) in the treated cells, sustaining the induced osteogenic program [60].

Although several studies demonstrated that a decrease in cellular stiffness after the depolymerization of actin filaments favors adipogenesis or chondrogenesis, whereas stimulation of actin polymerization enhances the osteogenic commitment of MSCs [21,23,24,25,26], probably a more complex cytoskeletal modulation drives the differentiation program. For example, it is evident that CB induces multiple effects (polymerization/depolymerization) on actin according to cell-type, as already demonstrated for CD [41]. In fact, CD was found to promote osteogenesis in bone marrow-derived stem cells (BM-MSCs) [61,62], whereas it either inhibited or enhanced this effect in human adipose-derived stem cells (hASCs) [63,64,65,66]. In this study, we have demonstrated that CB induced osteogenesis in hWJ-MSCs in association with an increased cell stiffness, while at the same concentration CB promoted adipogenesis in hASCs as reported in a previous manuscript of our research group [22]. These different results could be explained by a more complex role of actin in the regulation of MSC differentiation. In fact, the MSC fate is influenced by the balance between monomeric and polymeric actin in the cytoplasm and the concentration of nuclear actin, which affect the localization of relevant transcription factors [21].

## 4. Materials and Methods

### 4.1. Harvesting and Culture of hWJ-MSCs

Human WJ-MSCs were isolated from umbilical cords of 3 healthy donor mothers after informed consent as previously described [67]. The study was approved by the Local Ethical Committee of Bologna (IRCCS St. Orsola-Malpighi University Hospital Ethical Committee, protocol n° 2481/2017, ref n° 68/2017/U/Tess). Cells were cultured in Dulbecco’s Modified Eagle’s Medium—1 g/L of glucose (L-DMEM; Corning Incorporated, Corning, NY, USA) supplemented with 10% Fetal Bovine Serum (FBS; Gibco, Waltham, MA, USA) and antibiotics (1% Penicillin-Streptomycin Solution; Thermo-Fisher Scientific, Waltham, MA, USA) and were maintained in standard culture conditions at 37 °C with 5% carbon dioxide (CO_2_) in a humidified atmosphere. The non-adherent cells were removed, and medium was changed twice a week. When cells reached 80% confluence, they were detached using a trypsin-EDTA solution (Sigma-Aldrich Co., St. Louis, MO, USA) and were seeded for maintenance and/or expansion. For experiments, cells were seeded at a density of 5000 cells/cm^2^ (except where otherwise noted) in adequate plastic support (Corning Incorporated, Corning, NY, USA) and incubated in standard condition for 24 h before treatment. Experiments were performed using hWJ-MSCs at the 3rd–6th culture passages and were repeated in biological triplicate (*n* = 3).

### 4.2. hWJ-MSC Characterization

The hWJ-MSC phenotype and their trilineage differentiation potential were assessed.

To evaluate the expression of surface markers, cells were harvested using trypsin-EDTA, washed in Phosphate Buffered Saline (PBS, Sigma-Aldrich Co., St. Louis, MO, USA) and fixed with 4% formaldehyde (Sigma-Aldrich Co., St. Louis, MO, USA) for 10 min. Then, cells were incubated for 30 min at 4 °C in darkness with: CD90 and CD45 monoclonal antibodies (ABs) conjugated with fluorescein isothiocyanate (FITC); CD44 and CD14 monoclonal ABs conjugated with phycoerythrin (PE); CD105 and CD73 monoclonal ABs conjugated with phycoerythrin cyanin 7 (PC7); CD34 monoclonal AB conjugated with Peridinin Clorophyll (PerCP). All monoclonal ABs were purchased from eBioscienceTM (Thermo Fisher Scientific, San Diego, CA, USA). Cells were washed with PBS to remove unbound ABs. For each condition, a total of 10,000 viable cells (events) were acquired using the CytoFLEX S Flow cytometer (Beckman-Coulter Inc., Brea, CA, USA); data were analyzed by using the FlowJo v10.8 software (Tree Star, Ashland, OR, USA).

The osteogenic potential was evaluated as described in Section 4.13 of the “Materials and Methods” section.

In order to test the adipogenic potential, hWJ-MSCs were seeded at density 8000 cells/cm^2^ in 48-well plates (Corning Incorporated, Corning, NY, USA); when hWJ-MSCs reached confluence, adipogenesis was induced by “StemPro Adipogenesis Differentiation Kit” (Thermo Fisher Scientific, Waltham, MA, USA) following the manufacturer’s recommendations. Media were replaced twice a week for the entire induction protocol (14 days). At the end of the differentiation protocol, cells were washed in PBS, fixed in 4% formaldehyde for 45 min and stained with a filtered Oil Red O (O.R.O) solution (Sigma-Aldrich Co., St. Louis, MO, USA) 0.2% *w*/*v* in 60% 2-propanol (VWR International, Radnor, PA, USA) for 30 min at room temperature (RT). Cells differentiated into adipocytes showed red colored lipid vacuoles in cytoplasm.

To assess the chondrogenic potential, hWJ-MSCs were seeded at density 4000 cells/cm^2^ in 24-well plates (Corning Incorporated, Corning, NY, USA) and were cultured in standard conditions until they reached 80% confluence. Then, the standard medium was aspirated and replaced with complete StemPro Chondrocyte Differentiation Basal Medium (Thermo Fisher Scientific, Waltham, MA, USA). During 21 days of differentiation, the chondrogenic medium was changed every 3–4 days. The formation of cartilage proteoglycans was assessed by Alcian Blue staining (Sigma-Aldrich Co., St. Louis, MO, USA).

### 4.3. Cytochalasin B Treatments

Cytochalasin B (CB) was purchased from Sigma-Aldrich (Cytochalasin B from *Drechslera dematioidea*, Ready Made Solution 10 mg/mL in DMSO, Cat. number C2743; Sigma-Aldrich Co., St. Louis, MO, USA) and stored at −20 °C. CB was diluted at different concentrations in L-DMEM (range 0.01–5 μM). DMSO (the CB vehicle) treatment was included at the final concentration of 0.05%. Untreated cells were used as control (CTR).

### 4.4. Cell Count

hWJ-MSCs seeded in 6-well plates were exposed for 24 h to CB (range 0.01–5 μM) or DMSO. Each experimental condition was assayed in technical duplicate. At the end of the exposure-time, CTR and treated cells were detached by trypsin-EDTA and counted, as previously described [22]. Briefly, cells were resuspended in a medium with 50% of Erythrosine B red dye 0.2% (Sigma-Aldrich Co., St. Louis, MO, USA) in PBS. Not stained viable cells and red stained dead cells were manually counted, at least twice for each condition, using the Neubauer hemocytometer (BRAND GmbH, Wertheim, Germany) and a light microscope (Leica Labovert FS Inverted Microscope, Wetzlar, Germany). The total number of viable and dead cells was calculated according to the manufactured instructions.

### 4.5. Cell Death Detection

#### 4.5.1. Caspase-3 Expression

hWJ-MSCs were lysed using Mammalian Protein Extraction Reagent (M-PER, Thermo Fisher Scientific, San Diego, CA, USA) containing protease and phosphate inhibitors (Sigma-Aldrich Co., St. Louis, MO, USA). Protein concentrations were measured using Bradford Reagent (Sigma-Aldrich Co., St. Louis, MO, USA). Cellular lysates were resuspended in 4× Laemmli Sample Buffer and incubated at 95 °C for 5 min. Twenty micrograms of cellular lysates were separated to SDS-PAGE on a 10% Mini-PROTEAN^®^ TGX Stain-Free™ Precast Protein Gels (Bio-Rad Laboratories, Inc., Hercules, CA, USA) and transferred to a 0.2 µm nitrocellulose membrane (Bio-Rad Laboratories, Inc., Hercules, CA, USA) using the Trans-Blot^®^ Turbo™ Transfer System (Bio-Rad Laboratories, Inc., Hercules, CA, USA). After blocking, nitrocellulose membrane was incubated with caspase 3 primary antibody (1:1000 dilution; sc-7148 Santa Cruz Biotechnology Inc., Dallas, TX, USA) overnight at 4 °C, and then probed with horseradish peroxidase (HRP) conjugated secondary antibody (1:10,000 dilution; AbCam, Cambridge, UK) for 1 h at RT. Bound antibodies were detected with the use of Clarity Western ECL Substrate. The sample loading control was evaluated by a stain-free detection of proteins in the gel after electrophoresis.

#### 4.5.2. Annexin V Apoptosis Detection Assay

hWJ-MSCs seeded in 25 cm^2^ flasks (Corning Incorporated, Corning, NY, USA) were exposed for 24 h to CB (1 and 3 μM). The commercial FITC Annexin V Apoptosis Detection Kit with 7-Aminoactinomycin D (7-AAD) (640922; Biolegend, San Diego, CA, USA) was used to evaluate viability and cell death of hWJ-MSCs after CB-treatment for 24 h. hWJ-MSCs were washed twice in PBS and resuspended in Annexin V binding buffer at a concentration of 0.25–1.0 × 10^7^ cells/mL. The cell suspension (100 µL) was incubated with FITC Annexin V (5 µL) and 7-AAD Viability Staining Solution (5 µL) for 15 min at RT in darkness. Fluorescence was acquired using the CytoFLEX S Flow cytometer (Beckman-Coulter Inc., Brea, CA, USA) and data were analyzed by the FlowJo v10.8 software (Tree Star, Ashland, OR, USA). The scatterplots showed: in the lower left quadrant (Q4), living cells (which are negative for both Annexin V and 7-AAD markers); in the lower right quadrant (Q3), cells in the early-stage of apoptosis (labeled by FITC-conjugated Annexin V); in the upper left quadrant (Q1), cells in necrosis (labeled by 7-AAD); and in the upper right quadrant (Q2), cells in the late-stage of apoptosis which are positive for both markers.

### 4.6. Cell Proliferation Assay and Associated Cell Cycle Analysis

#### 4.6.1. Bromodeoxyuridine (BrdU) Assay

Bromodeoxyuridine (BrdU) assay (Sigma-Aldrich Co., St. Louis, MO, USA) was used to evaluate CB effects on cell proliferation. hWJ-MSCs, seeded on 96-well plates at density of 3000 cells/cm^2^, were exposed for 24 h and 72 h to CB (0.1, 1, and 3 μM) or DMSO. At the end of the exposure-time, according to the BrdU manufacturer’s instructions, cells (3 wells/conditions) were incubated with 20 μL/well BrdU labelling solution for 3 h at 37 °C, and then fixed with 200 μL/well FixDenat reagent for 30 min at RT. A negative control (medium without cells) was included. Afterwards, hWJ-MSCs were incubated with peroxidase conjugated anti-BrdU AB, and then with the peroxidase substrate. The reaction was stopped by adding 25 μL of H_2_SO_4_ 1M to each well. The absorbance at 450 nm was recorded with the Wallac 1420 Victor2 Multilabel Counter (Perkin Elmer, Waltham, MA, USA). hWJ-MSC proliferative ability was expressed as percentage of BrdU incorporated in treated cells compared to CTR cells (set to 100%) ± standard deviation (SD).

#### 4.6.2. Cell Cycle Progression and Gene Expression Analysis

To evaluate the influence of CB on the cell cycle progression, flow cytometry and gene expression analyses were performed. For flow cytometry analysis, hWJ-MSCs were seeded in 75 cm^2^ flasks (Corning Incorporated, Corning, NY, USA). After 24 h in standard conditions, cells were exposed to CB 1 μM or DMSO for 24 h. Untreated cells were used as CTR. At the end of treatment, hWJ-MSCs were detached from the plastic support and counted. Cells were washed once in 1X PBS and then, for each condition, 500,000 cells were fixed with 70% ice-cold ethanol overnight at −20 °C. Then, cells were washed with 1X PBS and incubated with a solution containing 50 µg/mL RNase for 30 min at RT. hWJ-MSCs were washed to remove RNase and then were stained with 50 µg/mL propidium iodine (PI). DNA content was analyzed using the CytoFLEX S Flow cytometer (Beckman-Coulter Inc., Brea, CA, USA); data were analyzed by using the FlowJo v10.8 software (Tree Star, Ashland, OR, USA).

To evaluate the expression of genes involved in proliferation (*MKI67*), cell cycle control (*CCND1*, *CDKN1A* and *CDKN2A*), and stemness (*OCT-4* or *POUF51A*), hWJ-MSCs seeded in 25 cm^2^ flasks (Corning Incorporated, Corning, NY, USA) and treated as above reported, were harvested for RNA isolation as described in Section 4.7.

### 4.7. RNA Extraction and RT-PCR

Total RNA was extracted using the RNeasy Mini Kit (QIAGEN, Valencia, CA, USA) and digested with RNase-free Deoxyribonuclease I (RNase-free DNase set—QIAGEN, Valencia, CA, USA) following the manufacturer’s instructions. RNA was reverse transcribed in cDNA (iScript™ RT Supermix; Bio-Rad Laboratories, Inc., Hercules, CA, USA). To verify whether the retrotranscription reaction was successful, *glyceraldehyde 3-phosphate dehydrogenase* (*GAPDH*) gene amplification and amplicon detection were performed as previously described [68,69].

### 4.8. Real-Time PCR

For each experimental condition, 25 ng of cDNA was amplified using the SsoAdvanced Universal SYBR Green Supermix (Bio-Rad Laboratories, Hercules, CA, USA) in technical triplicates using the Bio-Rad CFX96 real-time thermal cycler (Bio-Rad Laboratories, Hercules, CA, USA), as previously described [22,68]. The gene expression was determined by CFX Manager Software version 3.1 (Bio-Rad Laboratories, Hercules, CA, USA) using the “delta-delta CT method” [70]. To normalize the expression of gene involvement in cell cycle progression, proliferation and stemness (*MKI67, CCND1*, *CDKN1A*, *CDKN2A*, and *OCT-4*), three reference genes *GAPDH*, *TATA box binding protein* (*TBP), tyrosine 3 monooxygenase/tryptophan 5-monooxygenase activation protein zeta (YWHAZ)* were used.

For osteogenesis experiments, the expression of the specific osteogenic markers *RUNX2* and *BGLAP*, and of the autophagy related genes, *ATG7*, *LC3A,* and *BECN1*, was evaluated; *YWHAZ*, *TBP* and *hypoxanthine phosphoribosyl transferase 1* (*HPRT1*) were used as reference genes.

*GAPDH*, *TBP*, *HPRT1*, *CCND1*, *CDKN1A*, *CDKN2A,* and *OCT-4* primers were purchased from Bio-Rad (20X, Bio-Rad Laboratories, Hercules, CA, USA); all the other sequences were provided from Sigma-Aldrich (Sigma-Aldrich Co., St. Louis, MO, USA). The list of primer sequences was reported in Table 1. For each gene, the normalized expression value of untreated cells (CTR) or DMSO treated cells was set to 1, and all other gene expression values are reported to that value. Data are expressed as fold change ± SD.

### 4.9. Wound Healing Assay

For cell migration analysis, hWJ-MSCs were seeded into 24-well plates. After cells reached 100% confluence, a scratch, which consists of two straight lines that crossed in the middle of the well, was made by a pipette tip. Then, hWJ-MSCs were cultured for 24 h in absence or presence of CB (0.1, 1, and 3 µM). Images were acquired at 0 and 24 h from the scratch using a light microscope (Leica Labovert FS Inverted Microscope; Wetzlar, Germany) equipped with a Leica MC170 HD Imaging System Camera (Wetzlar, Germany). To evaluate the reversible effect of CB, after 24 h of treatment, the medium was replaced with standard fresh medium in all samples, and then cells were cultured for additional 24 h before images acquisition.

### 4.10. Morphological Analysis

hWJ-MSCs seeded at density of 3000 cells/cm^2^ in 24-well plates were exposed for 24 h to CB (0.1, 1 and 3 µM) or DMSO. Every experimental condition was assayed in technical duplicate. At the end of the treatment, a morphological analysis was performed, and representative images of cells were acquired under a light microscope (Leica Labovert FS Inverted Microscope; Wetzlar, Germany) with a Leica MC170 HD Imaging System Camera (Wetzlar, Germany).

### 4.11. Immunofluorescence of Cytoskeletal Markers

hWJ-MSCs were seeded in a 24-well plate containing glass coverslips (Corning Incorporated, Corning, NY, USA) at density of 6000 cells/cm^2^. After 24 h in standard conditions, hWJ-MSCs were treated for 24 h with CB (0.1, 1 and 3 µM) or DMSO. Each experimental condition was performed in technical duplicate. At the end of treatment, cells were fixed with 4% formaldehyde (Sigma-Aldrich Co., St. Louis, MO, USA) for 15 min, and then washed with PBS-Tween 0.25% (Sigma-Aldrich Co., St. Louis, MO, USA). Cells were permeabilized with Triton X-100 0.25% and sodium citrate 10 mM (Sigma-Aldrich Co., St. Louis, MO, USA) in PBS for 15 min at RT. Samples were blocked for 1 h with a solution containing 4% Bovine Serum Albumin (BSA; Sigma-Aldrich Co., St. Louis, MO, USA) and 0.3% Triton X-100 in PBS. To visualize focal adhesions and intermediate filament, cells were incubated with the primary ABs anti-vinculin (MAB3574; Chemicon-Sigma-Aldrich Co., St. Louis, MO, USA) or anti-vimentin (CS 5741; Cell Signaling Technology, Danvers, MA, USA), respectively, for 3 h at RT. Then, cells were incubated with the appropriate fluorescence-conjugated secondary AB (anti-mouse conjugated with Tetramethylrhodamine-5-(and 6)-isothiocyanate) for 1 h at RT. Primary and secondary AB were diluted in a solution containing 2% BSA (Sigma-Aldrich Co., St. Louis, MO, USA) and 0.15% Triton X-100 in PBS (dilution 1:200). Moreover, to visualize F-Actin hWJ-MSCs were stained with Phalloidin, Fluorescein Isothiocyanate Labeled (P5282; Sigma-Aldrich Co., St. Louis, MO, USA). Incubation with NucBlue^®^ Fixed Cell ReadyProbes^®^ Reagent (DAPI, Molecular Probes™, Life Technologies—Thermo-Fisher Scientific, Waltham, MA, USA) was used to counter-stain nuclei. All slides were mounted with antifade AF-400 (Immunological Sciences, Rome, Italy). The detection and acquisition of images was performed using Nikon Inverted Microscope Eclipse Ti2-E (Nikon Instruments, Melville, NY, USA) and a Digital Sight camera DS-Qi2 (Nikon Instruments, Melville, NY, USA) through the imaging software NIS-Elements.

### 4.12. Atomic Force Microscopy

To perform AFM analysis, hWJ-MSCs seeded in a 35 mm Petri dish (Corning Incorporated, Corning, NY, USA) were analyzed with the BioScope I microscope equipped with a Nanoscope IIIA controller (Veeco Metrology, Plainview, NY, USA). The measurement of mechanical properties, such as the elasticity and the viscosity, was conducted exploiting the force spectroscopy technique of the AFM. Silicon nitride cantilever equipped with a pyramidal tip and with nominal spring constants of 0.06 N/m and 0.24 N/m were used. The cantilever spring constant was calibrated using the thermal noise method [71,72].

hWJ-MSCs treated for 24 h with CB (0.1, 1 and 3 μM) or untreated (CTR) were analyzed considering viscoelastic models to describe the complete approach and retract portions of the force curve. To perform this analysis, we exploited Ting’s model using a procedure like the one presented in the work by Efremov et al. [73,74]. In Ting’s model, the linear viscoelastic theory is exploited, and a relaxation process has to be assumed for the sample. In this model, to account for specific indentation geometries, the elastic modulus of the sample must be modified by including hereditary integrals (functions that are based on the past history of the deformation process) that describe the relaxation process. Ting’s solution for a rigid pyramidal indenter on a viscoelastic material is obtained according to the formulas:

(1) $$\{\begin{array}{c} F\left(t,\delta \left(t\right)\right)=\frac{1.4906\tan\left(\theta \right)}{2\left(1-\nu^{2}\right)}\overset{t}{\underset{0}{\int }}E\left(t-\xi \right)\frac{\partial \delta^{2}}{\partial \xi }d\xi \;for\;0\leq t\leq t_{m}\\ F\left(t,\delta \left(t\right)\right)=\frac{1.4906\tan\left(\theta \right)}{2\left(1-\nu^{2}\right)}\overset{t_{1}\left(t\right)}{\underset{0}{\int }}E\left(t-\xi \right)\frac{\partial \delta^{2}}{\partial \xi }d\xi \;for\;t_{m}<t\leq t_{ind}\\ with:\overset{t}{\underset{t_{1}\left(t\right)}{\int }}E\left(t-\xi \right)\frac{\partial \delta }{\partial \xi }d\xi =0\;for:\;t_{m}<t\leq t_{ind} \end{array}$$

where *F* is the tip/sample force; *t_ind_* is the time of the entire indentation cycle (approach and retract phases); *t_m_* is the inversion time of the indentation cycle; *t*_1_ is a parameter needed to compensate for the sample relaxation during the retraction phase, and it is calculated by the expression reported in the above formula, and *E*(*t*) represents the Young’s modulus relaxation expression. Traditional relaxation processes such as the ones that can be described by the combination of Maxwell and Kelvin–Voigt elements could be used. However, we found that the process of cell indentation is better described by the power-law model. This model exploits just two cell parameters to describe cell rheology: cell elasticity (stiffness) and cell viscosity or fluidity (the power-law exponent). This model is equivalent to the superposition of infinite spring-dashpot couples connected to each other in parallel. Accordingly, for *E*(*t*) we adopted the following expression:

(2) $$E\left(t\right)=E_{0}{\left(\frac{t}{t{}^{\prime }}\right)}^{-\alpha }\;$$

where *t*′ corresponds to the sampling time of the force curve, and *E*_0_ is the instantaneous Young’s modulus of the sample. The adopted relaxation expression is known as the power-law rheology model, and it is the typically exploited model for AFM rheological measurements of cells [74]. Equation (2) is numerically solved and a least-squares error fitting procedure using the experimental values of the force is exploited to find the best values for the parameters *E*_0_ and α. To find the representative values of Young’s modulus (*E*_0_) and power-law exponent (α), a 16 × 16 force volume map on a 5 × 5 or 8 × 8 μm^2^ area was acquired for at least 10 cells in each condition. All the force curves of each map were averaged to obtain a single value for *E*_0_ and α for each cell. The distribution of all the Log*E*_0_ and α values from all the analyzed cells were fitted with Gaussian functions, and the values corresponding to the peak were obtained for each condition. All the force-curve analysis was performed with in-house software made using Python. The vertical scanning tip speed was 8 μm/s; the total z-scan displacement was between 2.5 μm and 3 μm. The cells were imaged while keeping the temperature constant at 37 °C.

### 4.13. Osteogenesis

The hWJ-MSC ability to differentiate toward the osteogenic fate was evaluated in the presence of CB for the entire period of the induction. Cells were seeded at 4000 cells/cm^2^ in 24-well or in 6-well plates for Alizarin Red S staining and gene expression analysis, respectively. For both evaluations, when cells reached 70% confluence, they were cultured in technical duplicate with standard (not induced) or StemPro Osteogenesis Differentiation medium (Thermo-Fisher Scientific, Waltham, MA, USA). For Alizarin Red S staining experiments, hWJ-MSCs were treated with DMSO or CB at 0.1, 1, and 3 μM and untreated cells were used as control (CTR); for Alizarin Red S staining and gene expression analysis, hWJ-MSCs were treated only with CB 1 μM and results were compared to those of DMSO-treated cells; both analyses were performed at 3, 7, 14, and 21 days from the beginning of the differentiation protocol. In all experiments, media were changed twice a week for 21 days.

The quantitative and qualitative assessment of osteogenic differentiation was performed by staining calcium deposits with Alizarin Red S solution after 21 days from the beginning of the differentiation protocol. Briefly, cells were fixed with 4% formaldehyde solution in PBS for 15 min and stained with Alizarin Red S solution 1% *w*/*v* (Sigma-Aldrich Co., St. Louis, MO, USA) in deionized water solution (pH = 4.1) for 15 min. The exceeding dye was removed by washing wells with deionized water. Images were acquired using the Leica Labovert FS Inverted Microscope (Wetzlar, Germany) with a Leica MC170 HD Imaging System Camera (Wetzlar, Germany). To quantify the amount of calcium deposits, the Alizarin Red S dye was solubilized with 10% acetic acid (Sigma-Aldrich Co., St. Louis, MO, USA); the solution was loaded in a 96-well plate in technical triplicate to measure the absorbance at 405 nm using a spectrophotometer plate reader (Wallac 1420 Victor2 Multilabel Counter; Perkin Elmer, Waltham, MA, USA). In parallel, for the gene expression analysis, cells were lysed at the chosen experimental time points for RNA extraction (see Section 4.7). Both Alizarin Red S staining and gene expression analysis were performed in biological triplicate (*n* = 3).

In addition, to confirm the relation between CB, the cytoskeleton changes, and osteogenesis process, the expression and distribution of F-Actin and osteopontin, were analyzed at 7 days from the beginning of the osteogenic protocol. hWJ-MSCs were cultured in osteogenic differentiation medium and were treated with CB 1 μM for the entire induction period or for only 24 h followed by CB washout. DMSO-treated cells were used as control. Immunofluorescence analysis was conducted as reported in Section 4.11, and specific primary AB anti-osteopontin (69498; AbCam, Cambridge, UK) was diluted at 1:200 in a solution containing 2% BSA (Sigma-Aldrich Co., St. Louis, MO, USA) and 0.15% Triton X-100 in PBS.

### 4.14. Statistical Analysis

Appropriate parametric tests (One-Way ANOVA and post-hoc Tukey Test) or nonparametric equivalents (Kruskal-Wallis test with post-hoc Dunn’s test) were used. Results are shown as mean ± SD. A *p* value < 0.05 was considered statistically significant.

## 5. Conclusions

In conclusion, our data demonstrated that CB influences in a dose-dependent manner hWJ-MSC properties, by acting at the nanomechanical level. Consequently, the tight relationship between CB-induced cytoskeletal disorganization and the modulation of osteogenic commitment of hWJ-MSCs paves the way for future studies to clarify the role of CB as a tool to control human stem cell properties, as well as their pluripotency, proliferation capacity, and specific commitment ability, thus favoring their clinical application in regenerative medicine.

## Figures and Tables

**Table 1:** Primer sequences of the genes analyzed by real-time PCR in hWJ-MSCs.

Gene	Entrez Gene ID *	Left Primer	Right Primer	Bio-Rad Unique Assay ID	A.L. (bp) ^$^
*Glyceraldehyde 3-phosphate dehydrogenase* *(GAPDH)*	2597	-	-	qHsaCED0038674	117
*TATA box binding protein* *(TBP)*	6908	-	-	qHsaCID0007122	120
*Hypoxanthine phosphoribosyl transferase 1* *(HPRT1)*	3251	-	-	qHsaCID0016375	90
*Tyrosine 3 monooxygenase/tryptophan 5-monooxygenase activation protein zeta* *(YWHAZ)*	7534	tcccgtttccgagccataaa	tgacctacgggctcctacaa	-	233
*Cyclin dependent kinase inhibitor 1A* *(CDKN1A or p21)*	1026	-	-	qHsaCID0014498	159
*Cyclin dependent kinase inhibitor 2A* *(CDKN2A or p16^INK4α^)*	1029	-	-	qHsaCED0056722	86
*Cyclin D1* *(CCND1)*	595	cagatcatccgcaaacacgc	aagttgttggggctcctcag	-	143
*Octamer-binding transcription factor 4 (OCT-4 or POUF51A)*	5460	-	-	qHsaCED0038334	100
*Proliferation marker protein Ki-67* *(MKI67)*	4288	tcagactccatgtgcctgag	ttgtcctcagccttctttgg	-	134
*RUNX family transcription factor 2* *(RUNX2)*	860	ctccctgaactctgcaccaa	tagagtggatggacggggac	-	149
*Bone gamma-carboxyglutamic acid-containing protein* *(BGLAP or Osteocalcin)*	632	caccgagacaccatgagagc	ctgcttggacacaaaggct	-	132
*Autophagy related 7* *(ATG7)*	10533	agcagctcatcgaaagccat	ttggcaaaaagcgatgagcc	-	241
*Microtubule associated protein 1 light chain 3 alpha* *(MAP1LC3A or LC3A)*	84557	ttggtcaagatcatccggcg	cctgggaggcgtagaccata	-	163
*Beclin 1* *(BECN1)*	8678	aaccagatgcgttatgccca	tccattccacgggaacactg	-	148

* ID: identification number; ^$^ A.L. (bp): Amplicon length (base pair).

## Data Availability

Data is contained within the article and Supplementary Material.

## Supplementary Materials

The following supporting information can be downloaded at: https://www.mdpi.com/article/10.3390/ph16020289/s1, Figure S1: TGX stain-free gel loaded with cell lysates of human Wharton’s jelly mesenchymal stem cells (hWJ-MSCs) after 24 h and 72 h of cytochalasin B (CB) treatment. Cellular lysates of untreated (CTR) and treated cells with CB 1 μM or dimethyl sulfoxide (DMSO) 0.05% (CB vehicle) were separated to SDS-PAGE on a 10% Mini-PROTEAN^®^ TGX Stain-Free™ Precast Protein Gels (Bio-Rad Laboratories, Inc., Hercules, CA, USA). Molecular weight marker: Precision Plus Protein Dual Color Standards (Bio-Rad Laboratories, Inc., Hercules, CA, USA); Figure S2: Immunofluorescence of human Wharton’s jelly mesenchymal stem cells (hWJ-MSCs) cytoskeletal markers after 24 h of cytochalasin B (CB) treatment. hWJ-MSCs were immunostained with Phalloidin (grey signal, specific for F-Actin), anti-vimentin (green signal), and anti-vinculin (red signal) after 24 h of CB-treatment. Images were acquired with a Nikon inverted microscope Eclipse Ti2-E and a digital sight camera DS-Qi2, through the imaging software NIS-Elements. Images refer to cells treated for 24 h with CB 3 μM. White arrows indicate cortical actin (upper panel), perinuclear vimentin (middle panel), and binucleated cells (lower panel). Scale bars: 50 μm. Figure S3: Cell viability of human Wharton’s jelly mesenchymal stem cells (hWJ-MSCs) after 3, 7, 14, or 21 days of cytochalasin B (CB) treatment. T: time; 3, 7, 14, or 21 days: after 3, 7, 14, or 21 days of treatment; CB: cytochalasin B-treated cells; DMSO: dimethyl sulfoxide-treated cells; CTR: control (untreated cells). Cell viability: percentage of living cells/total number of cells ± standard deviation (SD).
