# Supplementary figures and images for: Cytochalasin B Influences Cytoskeletal Organization and Osteogenic Potential of Human Wharton’s Jelly Mesenchymal Stem Cells
Source: Pharmaceuticals (Basel). 2023 Feb 14;16(2):289. doi: 10.3390/ph16020289 (PMC9966134; doi:10.3390/ph16020289)

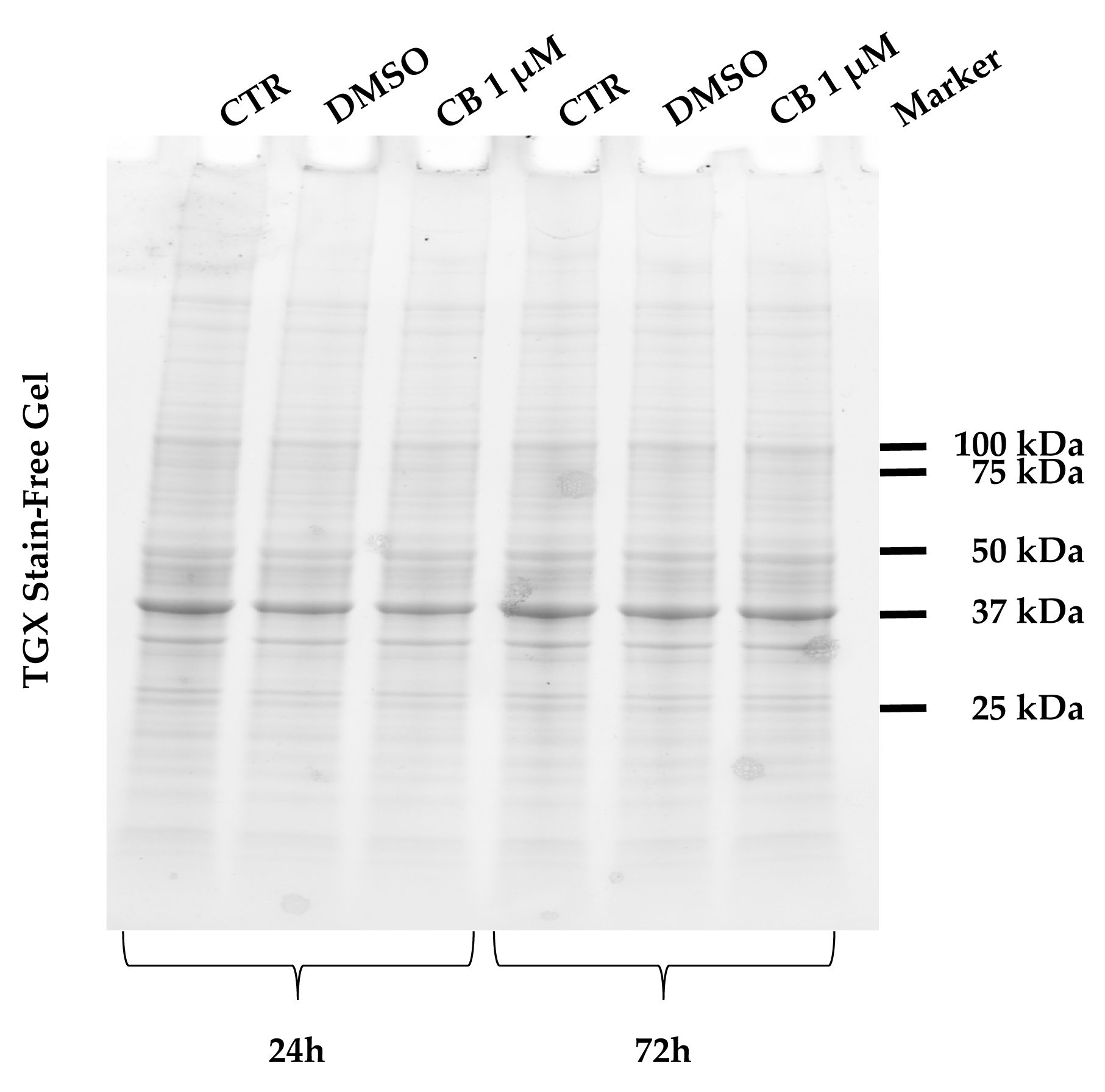

Supplement: Supplementary file 1 [file pharmaceuticals-16-00289-s001.zip › Supplementary materials/Figure S1.jpg]

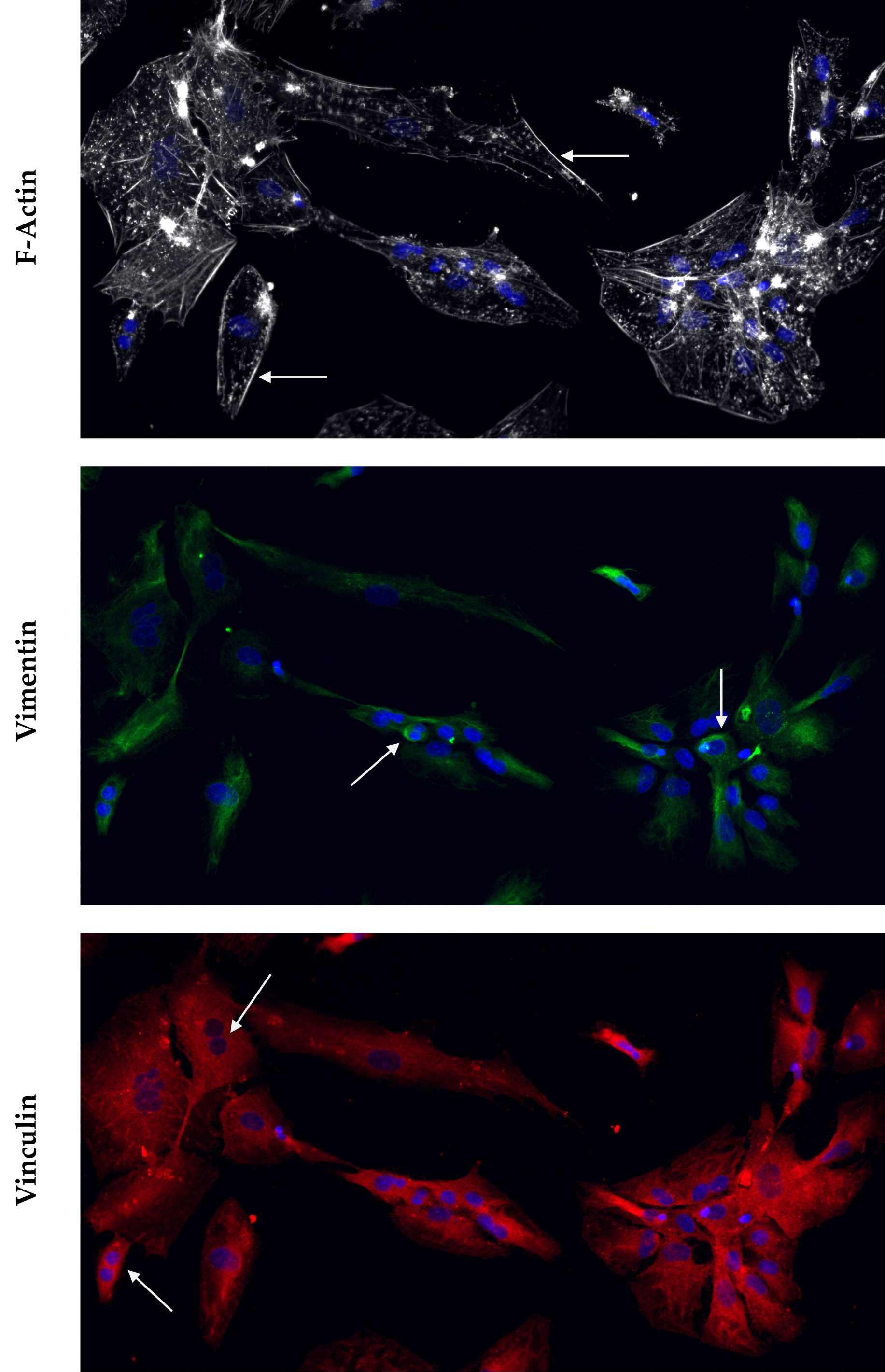

Supplement: Supplementary file 1 [file pharmaceuticals-16-00289-s001.zip › Supplementary materials/Figure S2.jpg]

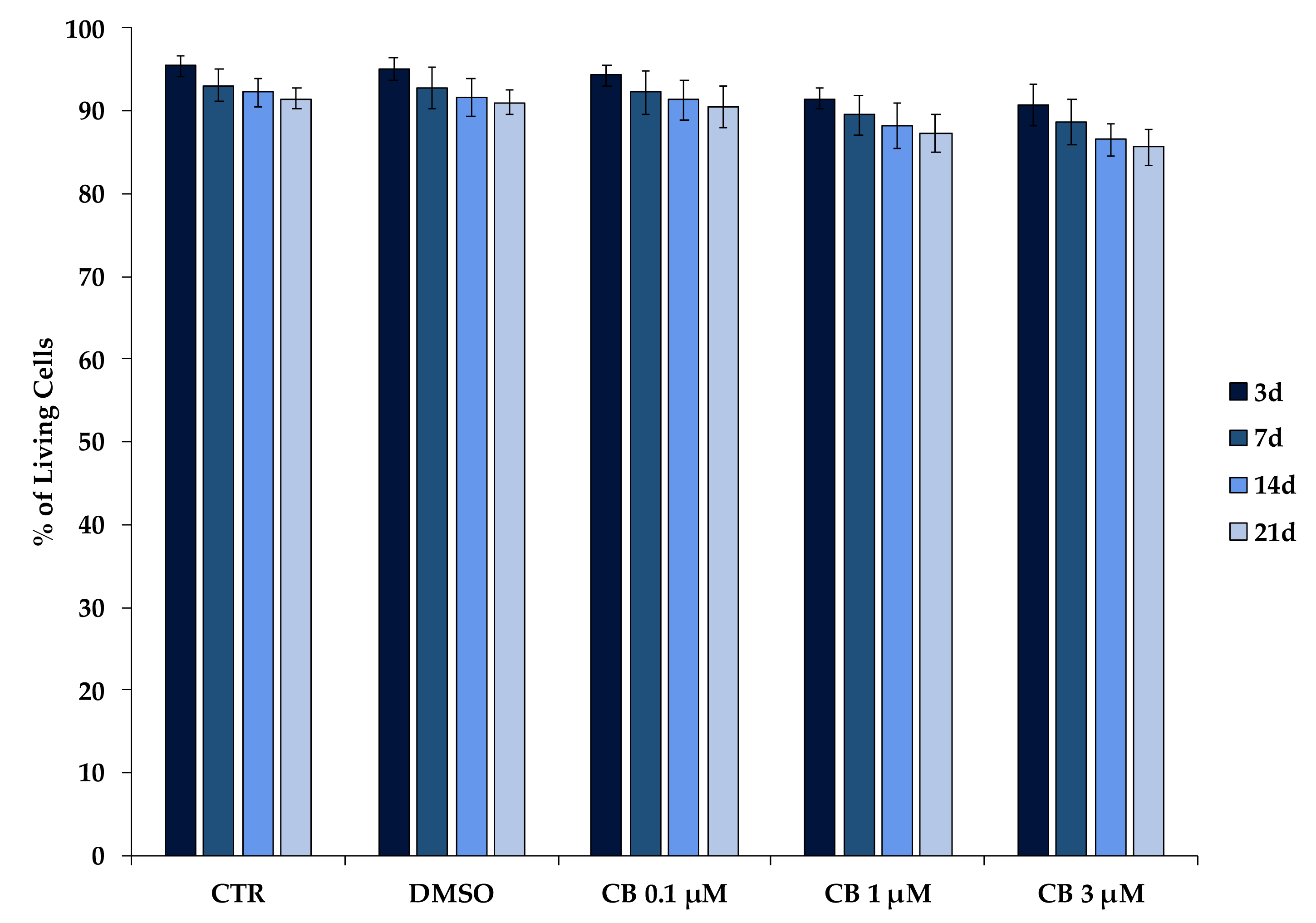

Supplement: Supplementary file 1 [file pharmaceuticals-16-00289-s001.zip › Supplementary materials/Figure S3.jpg]
